# Changes of the erythrocyte phenotype and blood biochemistry in dairy calves during the first ten weeks of age

**DOI:** 10.7717/peerj.7248

**Published:** 2019-07-16

**Authors:** Lennart Golbeck, Imke Cohrs, Theresa Scheu, Walter Grünberg

**Affiliations:** 1 Clinic for Cattle, University of Veterinary Medicine Hannover, Foundation, Hanover, Germany; 2 Hofgut Neumühle, Governmental Institute for Education and Research, Münchweiler an der Alsenz, Germany

**Keywords:** Iron, Potassium, Sodium, Microcytosis, Anemia

## Abstract

**Background:**

Bovine erythrocytes undergo important changes in their morphology and chemical composition during the first weeks of age, which must be understood to accurately interpret hematology results in calves. The objectives of this prospective cohort study were to describe physiological changes of calf erythrocytes and to investigate mechanisms potentially causing these changes.

**Methods:**

Blood samples from 30 clinically healthy dairy calves were obtained from birth to the tenth week of age in weekly intervals. Hematological and plasma biochemical parameters as well as the mineral electrolyte content of erythrocytes were determined and followed over time. The changes of parameters characterizing the erythrocyte phenotype over time were compared to the changes of plasma and erythrocyte biochemical parameters and possible associations were investigated using correlation and stepwise regression analyses.

**Results:**

Although the erythrocyte mean corpuscular volume (MCV) declined from 43.6 ± 3.7 fL to 35.6 ± 3.2 fL between the first and seventh week, the red blood cell count (RBC) increased from 7.2 ± 1.1 × 10^12^/L to 9.3 ± 1.0 × 10^12^/L until the fifth week of age. The blood hemoglobin (Hb) concentration increased from 0.96 ± 0.16 g/L to 1.16 ± 0.11 g/L in the first three weeks of age and remained at this level until the end of the study. Changes in MCV were accompanied by a decline of the erythrocyte potassium content (K_ERY_) from 91.9 ± 13.5 to 24.6 ± 7.2 mmol/L and a concomitant increase of the erythrocyte sodium content from 45.0 ± 32.0 to 102.7 ± 26.5 mmol/L. MCV was found to be associated with K_ERY_, the primary determinant of the intra-erythrocyte osmotic pressure from the sixth week of age and with blood hemoglobin, the primary determinant of the intra-erythrocyte oncotic pressure from the eighth week of age, when K_ERY_, blood Hb and MCV already had reached or approached normal levels of adult cattle. The plasma iron concentration was not found to be associated to any of the studied hematological parameters.

**Conclusion:**

A volume reduction of 20% in bovine neonatal erythrocytes is a physiological change occurring during the first weeks of age and is neither associated with sideropenia nor with anemia in healthy calves. The mechanism driving the observed erythrocyte volume change could not be identified. Results of the correlation and regression analyses indicate that changes in intra-erythrocyte osmotic or oncotic pressure are improbable underlying causes. Results reported here show that K_ERY_ is an unreliable indicator for the K homeostasis of the intracellular space in neonatal calves and that a decrease in MCV in early life per-se is an unreliable indicator for the development of microcytic anemia.

## Introduction

Laboratory diagnostic tests can present a valuable tool for the production animal veterinarian to monitor herd health or to accurately diagnose early stage disease in individual animals. The meaningful and efficient use of laboratory results in calves requires a good understanding of physiological changes occurring as the animal grows. To improve the diagnostic precision of cell counts, cell indices or blood biochemical parameters, the establishment of reference intervals specific for age, gender, breed or production type has been encouraged in the literature (Dillane et al., 2018; Mohri, Sharifi & Eidi, 2007; Moretti et al., 2017; Panousis et al., 2018; Shaffer, Roussel & Koonce, 1981). Hematologic parameters of bovines such as the erythrocyte count, cell volume or the biochemical composition of erythrocytes are subject to important changes during the first months of life. Changes occurring in juvenile bovine erythrocytes include the replacement of fetal by adult hemoglobin (Hb), a reduction of the mean corpuscular volume (MCV), a reduction of the intracellular concentration of potassium ([K]) and the concomitant increase of the intracellular sodium concentration ([Na]) (Grimes, Duncan & Lassiter, 1958; Israel et al., 1972; Mohri, Sharifi & Eidi, 2007). Investigating these changes that occur in the first weeks of age is of interest as some parameters are used for example to diagnose metabolic disturbances of the iron homeostasis in calves, or potassium depletion in sick neonates (Bostedt et al., 2000; Bostedt, Jekel & Schramel, 1990; Golbeck et al., 2018; Volker & Rotermund, 2000; Wittek, Köchler & Mader, 2014). A decline of the MCV that physiologically occurs in early life is also considered a hallmark of iron deficiency anemia. For the accurate interpretation of laboratory results it is thus essential to differentiate between physiological and pathological changes (Constable et al., 2017). Exploring associations between some of these parameters may furthermore provide valuable clues for a better understanding of mechanisms through which such changes take place. The objectives of this study thus were to describe hematological and specific blood biochemical changes occurring in healthy Holstein-Friesian calves during the first 10 weeks of age, and to identify associations between these parameters possibly explaining underlying mechanisms.

## Materials and Methods

### Animals

This study was approved by the Animal Welfare and Ethics Committee of the government of Coblenz, Rhineland Palatinate, Germany (permit no A 17-20-001 OEW), and conceived as a prospective observational cohort study on clinically healthy Holstein-Friesian dairy calves covering the period from birth to the tenth week of age and reared under field conditions. The study was conducted between September 2017 and February of 2018 and included 25 female and five male calves.

### Housing and feeding

Calves were housed individually in calf hutches bedded with straw from birth to approximately 14 days of age, and where thereafter moved to indoor group pens with up to 15 calves per pen, separated by gender. Immediately after birth calves were separated from their dam and fed a minimum of 3 L of colostrum within the first two hours of life. Calves not ingesting this minimum amount voluntarily in the allotted time were force-fed the remaining volume of colostrum with a stomach tube. Calves received colostrum from their respective dams whenever the quality as assessed by colostrometry was adequate. In cases of inadequate quality, thawed colostrum of confirmed quality previously stored at −20 °C was administered. Immediately following colostrum intake, calves were administered 2 mL of an iron supplement orally containing 230 mg of Fe^3+^, 10000 IE Vitamin A and 100 mg of Vitamin E (Sinta® Fer-o-Bac; Sinta GmbH, Schwarzenborn, Germany) per dose. This was a standard procedure at the research farm with the objective to prevent a latent iron deficiency in early life.

During the first five days of life 4 L of acidified whole milk were offered twice daily from a nipple bucket. For this purpose, a commercial milk acidifier (ascorbic acid, lignosulfonic acid, lactic acid, ammonium formate, ammonium propionate, Schaumacid Drink, H. Wilhelm Schaumann GmbH & Co. KG, Brunn/Gebirge, Austria) was added to milk (2 mL/L) to reach a whole milk pH of 5.5. Thereafter calves were switched to 4 L of milk replacer (MR, 14% dry matter (DM)) twice daily, using a commercial MR powder (22% crude protein, 18% crude fat, 100 mg/kg Fe as ferrous chelate of glycine; Milkibeef Plus, Milkivit, Trouw Nutrition Deutschland GmbH, Burgheim, Germany) that was also offered from a nipple bucket. In the group pens MR was offered through an automated calf feeding system dispensing up to 2 L per feeding. Heifer calves received 8 L of MR per day throughout the study while bull calves were offered 10 L of MR from two weeks of age. Daily consumption of whole milk and MR was recorded. Hay and clean water offered from a bucket were available ad libitum starting from two days of age. Calf pellets (Blattina Trocken TMR, Blattin Mineralfutterwerk Seitschen GmbH & Co. KG, Göda, Germany) were offered starting at 14 days of age. From 60 days of age on heifer calves were gradually weaned over the course of 10 days. Weaning of bull calves was started at the same age but was extended over more than 10 days due to higher daily milk consumption.

### Standard procedures

Shortly after birth calves underwent a complete clinical examination to confirm the suitability for inclusion in this study, and were weighed on an electronic scale. Inclusion criteria were clinical health at birth as determined by one investigator (T.S.) and female gender of the calf. Gender selection was necessary because bull calves routinely left the research farm at two weeks of age. To be able to identify a possible gender effect on any of the studied parameters a small number of male calves were also selected for inclusion. As gender specific differences have not been reported for any of the key parameters of this study, data on which to base a power analysis were not available. A number of 5 male calves to be included in this study was arbitrarily determined based on availability and practicability on the experimental farm.

Blood samples were obtained once a week at a standardized weekday and time of the day. Blood sampling was always preceded by a physical examination. Blood was obtained by venipuncture of a jugular vein with a hypodermic needle (18 G; Henry Schein Inc., Melville, NY, USA), and collected in lithium heparin- and K_3_-EDTA tubes (10 mL Li Heparin and 4 mL EDTA K; Sarstedt AG & Co. KG, Nürnbrecht, Germany).

### Sample processing and analysis

K_3_-EDTA blood tubes were kept at room temperature and were analyzed within 24 h of sample collection on an automated hematology analyzer (Advia 120 Hematology System; Siemens Healthcare GmbH, Erlangen, Germany) to determine the red blood cell count (**RBC**), the mean corpuscular volume (**MCV**) and the hemoglobin concentration (**Hb**). The hematology analyzer furthermore calculated the packed cell volume (**PCV**) as well as the erythrocyte indices mean corpuscular hemoglobin content (**MCH**) and mean corpuscular hemoglobin concentration (**MCHC**).

Blood in lithium-heparin tubes was centrifuged at 3500xg for 10 min at room temperature within 10 min of sample collection; plasma was harvested and immediately frozen at −20 °C (−4 °F). Plasma **[K]** and **[Na]** were measured by indirect potentiometry (ABX Pentra 400, Horiba Europe GmbH, Langenhagen, Germany). Samples obtained at weeks 1, 4, 7 and 10 were assayed for their plasma iron concentration ([Fe]) by inductively coupled plasma optical emission spectrometry (ICP-OES).

A hemolysate of whole blood was prepared by thoroughly mixing 0.5 mL of lithium heparinized whole blood with 2.5 mL of deionized water, and stored at −20 °C (−4 °F) until analyzed for the concentration of potassium ([K]) and sodium ([Na]) by ICP-OES.

Erythrocyte K and Na contents (in mmol/L erythrocytes, K_ERY_ and Na_ERY_ respectively) were derived from [K] and [Na] in plasma and hemolysate (mmol/L), and the PCV (L/L) using following equation:

\begin{eqnarray*}{\mathrm{K}}_{\mathrm{ERY }} \mathrm{or} {\mathrm{Na}}_{\mathrm{ERY }} \left( \frac{\mathrm{mmol}}{\mathrm{L}} \right) = \frac{ \left( \left( \mathrm{hemolysate} \left[ \mathrm{K} \right] \mathrm{or} [\mathrm{Na}]\ast 6 \right) - \left( 1-\mathrm{PCV } \right) \ast \mathrm{plasma} \left[ \mathrm{K} \right] \mathrm{or} [\mathrm{Na}] \right) }{\mathrm{PCV }} . \end{eqnarray*}

The factor 6 was used to account for dilution of the hemolysate with deionized water. A similar equation has been described and used in an earlier study (Evans, 1957; Golbeck et al., 2018).

The intracellular cation concentration (**ICC**, mEq/L erythrocytes) was calculated as a crude indicator for the intracellular osmotic pressure:

\begin{eqnarray*}\mathrm{ICC} \left( \frac{\mathrm{mEq}}{\mathrm{L}} \right) ={\mathrm{K}}_{\mathrm{ERY }}+{\mathrm{Na}}_{\mathrm{ERY }}. \end{eqnarray*}

The extracellular cation concentration (**ECC**, mEq/L) as an indicator for the extracellular osmotic pressure was calculated as follows:

\begin{eqnarray*}\mathrm{ECC} \left( \frac{\mathrm{mEq}}{\mathrm{L}} \right) =\mathrm{plasma} \left[ \mathrm{K} \right] +\mathrm{plasma} [\mathrm{Na}]. \end{eqnarray*}

The difference (**DIFF**) between ECC and ICC as an indicator for the equilibrium between intra- and extracellular osmotic pressure was then derived:

\begin{eqnarray*}\mathrm{DIFF}=\mathrm{ECC}-\mathrm{ICC}. \end{eqnarray*}

Samples obtained at the time of disease were carefully evaluated. Samples with one or more parameters deviating more than 5% from the mean value of the corresponding week without the sample in question were excluded from statistical analyses.

### Statistical analysis

Data are presented as mean ± SD. Normal distribution was assessed with the Shapiro–Wilk test and the visual inspection of QQ plots.

Repeated measures analyses of variance with an autoregressive(1) covariance matrix, with animal ID as repeated subject were conducted to identify time-, gender- and time × gender interaction effects. The autoregressive(1) covariance structure was chosen based on the lowest Akaike information criterion. Bonferroni-adjusted *P* values were used to assess differences between male and female calves at specific sampling times whenever the *F* test was significant.

Pairwise Pearson’s correlation analyses were conducted to identify associations between RBC, Hb, MCV, K_ERY_, plasma [K] and plasma [Fe] stratified by week of age.

Multiple stepwise regression analyses were conducted to identify associations of MCV, Hb and K_ERY_ (dependent variables) with any of the other studied parameters (independent variables). Parameters were included as independent variables in the stepwise regression analysis with a *P* = 0.2 as entry and *P* = 0.05 as exit. These regression analyses were conducted including all sampling times in one analysis as well as stratified by sampling time. For the analyses including all sampling times a dummy variable coding was used to account for repeated measures. This approach that enforces a uniform slope but varying intercept values per calf is appropriate to account for inter-individual variation when slope and curve shape of included individuals are similar, as in the present study (Glantz & Slinker, 1990). Dummy variables (C_1_ to C_n_) were defined as follows: C_1_ = 1 if calf i (*i* < *n*), −1 if calf = n, and 0 otherwise. The final regression models were checked for variance inflation by screening tolerance, variance inflation factors, and the eigenvalue in combination with the condition index of each variable. In case of suspected multicolinearity between some of the variables in the final model, the variable potentially affected by colinearity with the lowest *R*^2^ was removed and the analysis rerun.

A *P* value of <0.05 was considered significant. A statistical software package was used for the statistical analysis (SAS 9.4; The SAS Institute, Cary, NC, USA).

## Results

Average birth weight of experimental animals was 40.7  ± 4.5 kg (mean  ± SD). Only minor disease incidents were recorded during the study. Therapeutic intervention was required in the first and second week for two calves with mild omphalitis, for two calves in the seventh week, one calf in the ninth week for respiratory tract disease, and two calves in the fifth and seventh week that developed mild diarrhea. None of the calves required treatment for more than one condition or on more than one occasion during the study. Treatment for omphalitis and respiratory tract disease consisted of parenteral antimicrobial therapy (Amoxicillin, 10 mg/kg BW subcutaneously) in combination with a non-steroidal anti-inflammatory drug (Meloxicam, 0.5 mg/kg BW subcutaneously). Mild diarrhea was exclusively treated with a non-steroidal anti-inflammatory drug (Metamizole, 20 mg/kg BW intramuscularly). Treated animals made uneventful recoveries in all instances. All samples obtained at the time of disease remained in the dataset for statistical analysis as none of the parameters deviated more than 5% of the mean value of the sampling week without the sample in question. Due to clotting, 10 EDTA blood samples could not be analyzed. Intake of MR/acidified whole milk was consistently equivalent to the total amount of liquid nutrient offered throughout the study. Transient feed intake depression was recorded on the day calves were moved from hutches to group pens, which was primarily attributed to the change of feeding system and stress from relocation/grouping (Fig. S1).

Gender specific differences were not apparent for any of the studied parameters.

**Figure 1:**
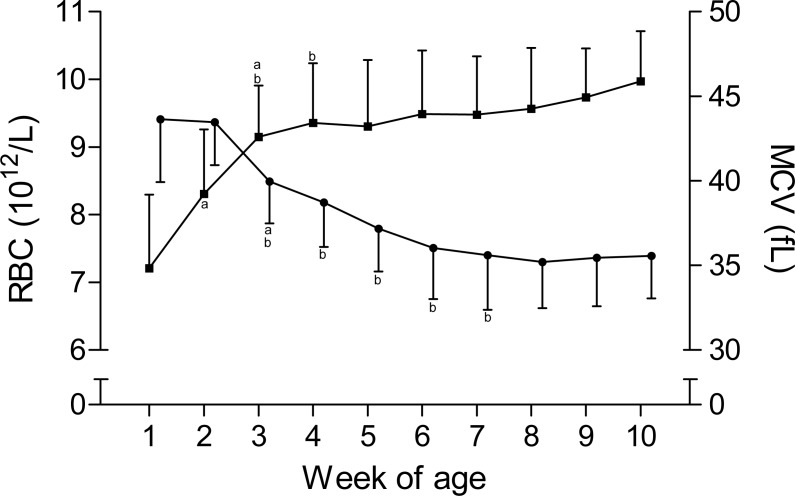
Red blood cell count and mean corpuscular volume in Holstein-Friesian calves over the course of the first 10 weeks of age. Mean (+SD) ** red blood cell count** (RBC, closed squares) and **mean corpuscular volume** (MCV, closed circles) in 30 Holstein-Friesian calves over the course of the first 10 weeks of age (*n* = 30 with exception of weeks 4, 6 and 9 *n* = 29, and week 3 with *n* = 28). Values marked with the superscript letter “a” differ significantly (*P* < 0.05) from the value of the preceeding week, whereas a “b” indicates a difference to the value of two weeks before.

**Figure 2:**
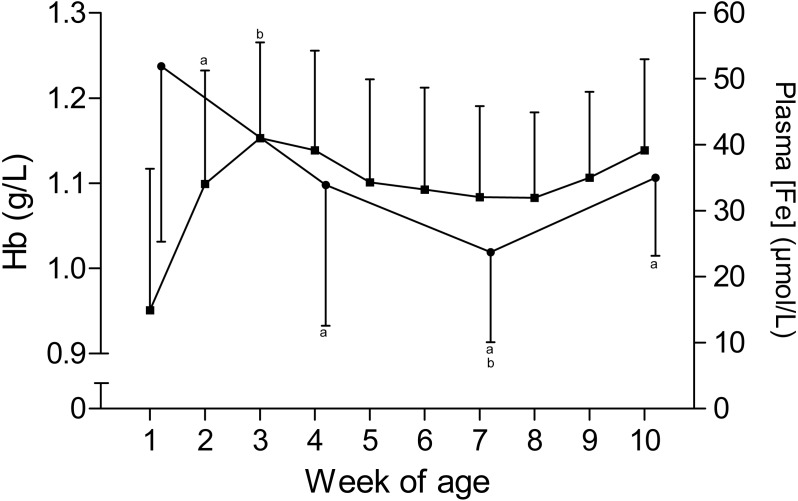
Hemoglobin and plasma iron concentration in Holstein-Friesian calves over the course of the first 10 weeks of age. Mean (+SD)** hemoglobin**(Hb, closed squares) and **plasma iron concentration** (plasma [Fe], closed circles) in 30 Holstein-Friesian calves over the course of the first 10 weeks of age (Hb: *n* = 30 with exception of weeks 4, 6 and 9 *n* = 29, and week 3 with *n* = 28; plasma [Fe]: *n* = 30 with exception of week 4 *n* = 29). Values marked with the superscript letter “a” differ significantly (*P* < 0.05) from the value of the preceeding week, whereas a “b” indicates a difference to the value of two weeks before.

The time curves for RBC and MCV are presented in Fig. 1. While the RBC increased from 7.2 ± 1.1 × 10^12^/L to 9.3 ± 1 × 10^12^/L in the first five weeks of age and to 10 ± 0.7 × 10^12^/L until the end of the study, the MCV declined from 43.6 ± 3.7 fL to 35.6 ± 3.2 fL between the first and seventh week, but remained stable thereafter. This inverse pattern for RBC and MCV resulted in a constant PCV throughout the study (Table 1). The blood hemoglobin concentration increased from 0.96 ± 0.16 g/L to 1.16 ± 0.11 g/L during the first three weeks of age, without any further change during the remainder of the study period (Fig. 2). Highest plasma [Fe] were measured in the first week of age that then declined continuously, reaching a nadir at seven weeks of age. Thereafter an increase in mean plasma [Fe] was observed until the end of the study (Fig. 2; Fig. S3). Values for plasma [Fe] were highly variable within and between animals. Plotting the plasma [Fe] values measured in the first week of age against the age (in days) at sampling, revealed a decrease in plasma [Fe] during the first week of age (Fig. S3).

**Table 1:** Selected blood hematological and biochemical parameters in 30 Holstein-Friesian calves over the course of the first 10 weeks of age.

Week	Plasma [K] (mmol/L)	Plasma [Na] (mmol/L)	PCV (L/L)	MCH (pg)	MCHC (g/dL)
1	4.7 ± 0.3	137.8 ± 3.2	0.31 ± 0.05	13.4 ± 1.2	30.6 ± 1.4
2	4.7 ± 0.2	137.7 ± 1.9	0.36 ± 0.04^a^	13.4 ± 0.8	30.8 ± 1.1
3	4.6 ± 0.4	138.3 ± 2.4	0.37 ± 0.03^b^	12.6 ± 0.6^a,b^	31.7 ± 0.9^b^
4	4.4 ± 0.3^a^	138.6 ± 2.0	0.36 ± 0.04	12.4 ± 0.7^b^	31.8 ± 1.1
5	4.3 ± 0.2	138.9 ± 2.6	0.34 ± 0.04^a,b^	11.9 ± 0.8^b^	32.1 ± 1.1
6	4.4 ± 0.3	138.4 ± 1.7	0.34 ± 0.03^b^	11.7 ± 0.7^b^	32.4 ± 1.4
7	4.4 ± 0.3	138.6 ± 2.2	0.34 ± 0.04^b^	11.6 ± 0.8	32.4 ± 1.5^b^
8	4.4 ± 0.4	138.8 ± 3.2	0.34 ± 0.03	11.5 ± 0.8	32.4 ± 1.4
9	4.3 ± 0.3^a^	138.9 ± 2.1	0.34 ± 0.03	11.5 ± 0.8	32.3 ± 1.0^a^
10	4.4 ± 0.3	139.9 ± 2.6	0.35 ± 0.03	11.5 ± 0.8	32.2 ± 1.4^b^

**Notes.**

Mean (±SD) of packed cell volume (PCV), mean corpuscular hemoglobin (MCH), mean corpuscular hemoglobin concentration (MCHC), plasma sodium (plasma [Na]) and plasma potassium concentration (plasma [K]) in 30 Holstein-Friesian calves over the course of the first 10 weeks of age.

Values marked with the superscript letter “a” differ significantly (*P* < 0.05) from the value of the preceeding week, whereas a “b” indicates a difference to the value of two weeks before. *n* = 30 except for plasma [Na] and PCV in week three (*n* = 28) and PCV in weeks four, six and nine (*n* = 29).

**Figure 3:**
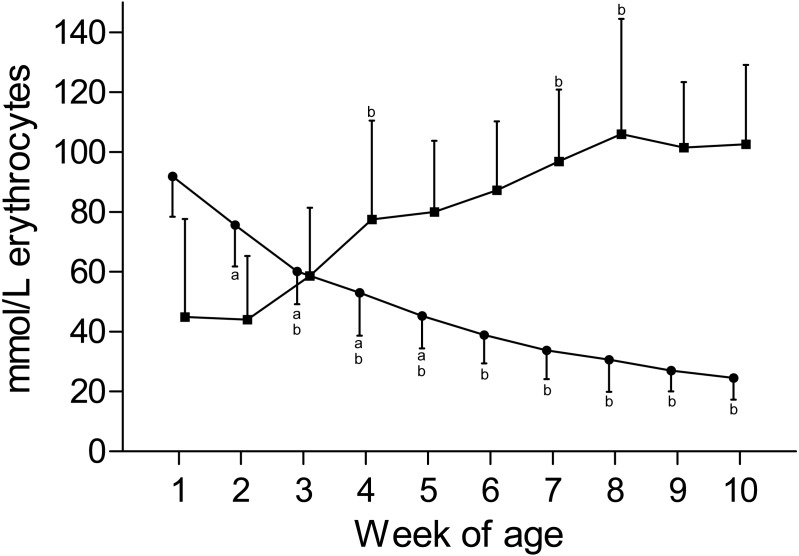
Erythrocyte potassium and sodium concentration in Holstein-Friesian calves over the course of the first 10 weeks of age. Mean (+SD) **K**_**ERY**_ (closed circles) and **Na**_**ERY**_ (closed squares) in 30 Holstein-Friesian calves over the course of the first 10 weeks of age (for both: *n* = 30 except for weeks 4, 6 and 9 with *n* = 29, week 3 with *n* = 28; for Na_ERY_: week 1 with *n* = 26). Values marked with the superscript letter “a” differ significantly (*P* < 0.05) from the value of the preceeding week, whereas a “b” indicates a difference to the value of two weeks before. Values for Na_ERY_ and K_ERY_ are slightly offset for better readability of the graph.

K_ERY_ and Na_ERY_ are depicted in Fig. 3. K_ERY_ declined from a peak value of 91.9 ± 13.5 mmol/L in the first week to a nadir of 24.6 ± 7.2 mmol/L at the end of the study. A concomitant increase of Na_ERY_ was observed from the mean nadir of 45.0 ± 32.0 mmol/L in the first week of age to a mean peak value of 106.1 ± 38.4 mmol/L measured at the eighth week of age. Although the change of Na_ERY_ over time mirrored the content-time curve of K_ERY_, a larger degree of variation was observed for Na_ERY_. Compared to the intracellular electrolyte contents, plasma [K] showed only a mild decline over time, and changes in plasma [Na] were insignificant (Table 1). This observation also reflects in calculated ECC values that remained constant over time. In contrast the ICC and thereupon the DIFF showed a larger degree of variation and subtle changes between weeks (Fig. S2).

The stepwise regression analysis with MCV as dependent variable including all sampling time points showed strongest associations with K_ERY_ (partial *R*^2^ = 0.480; *P* < 0.0001). The correlation analysis stratified by sampling week revealed significant negative associations of the MCV with K_ERY_ during the last five weeks of the study, and a positive association with Hb during the last two weeks.

The stepwise regression analysis with Hb as dependent variable including all time points showed strongest associations with RBC (partial *R*^2^ = 0.280; *P* < 0.0001). The regression coefficient however decreased over time in the analysis stratified by week of age (Table 2). The correlation analysis identified a negative association between Hb and K_ERY_ during the first and last two weeks of the experimental period, and a positive association between RBC and Hb over the entire study period (Table 3).

**Table 2:** Stepwise regression analysis for the associations with MCV, Hb, K_ERY_ and PCV as dependant variables, also stratified by week. Data from 30 healthy calves over the first 10 weeks of age.

Week	**MCV**	**Hb**	**K**_ERY_	**PCV**
	Stepwise regression analysis including all sampling times			
	K_ERY_:part. *R*^2^ = 0.480^***^	RBC:part. *R*^2^ = 0.280^***^	Na_Ery_:part. *R*^2^ = 0.076^***^	Hb:part. *R*^2^ = 0.368^***^
	DIFF:part. *R*^2^ = 0.021^*^	MCV:part. *R*^2^ = 0.120^***^	ICC: part. *R*^2^ = 0.022^***^	
	Stepwise regression analysis stratified by week of age			
1	NA	RBC:part. *R*^2^ = 0.756^***^	ICC:part. *R*^2^ = 0.553^***^	Hb:part. *R*^2^ = 0.945^***^
2	NA	RBC:part. *R*^2^ = 0.805^***^	Na_Ery_:part. *R*^2^ = 0.763^***^	Hb:part. *R*^2^ = 0.909^***^
			ICC:part. *R*^2^ = 0.237^*^	
3	NA	RBC:part. *R*^2^ = 0.767^***^	ECC:part. *R*^2^ = 0.165^*^	Hb:part. *R*^2^ = 0.894^***^
4	NA	RBC:part. *R*^2^ = 0.759^***^	DIFF:part. *R*^2^ = 0.559^***^	Hb:part. *R*^2^ = 0.888^***^
5	NA	RBC:part. *R*^2^ = 0.747^***^	ECC:part. *R*^2^ = 0.338^**^	Hb:part. *R*^2^ = 0.919^***^
6	Hb:part. *R*^2^ = 0.417^***^	RBC:part. *R*^2^ = 0.710^***^	MCV:part. *R*^2^ = 0.295^*^	Hb:part. *R*^2^ = 0.849^***^
	K_ERY_:part. *R*^2^ = 0.295^*^			
7	K_ERY_:part. *R*^2^ = 0.270^*^	RBC:part. *R*^2^ = 0.621^***^	Na_Ery_:part. *R*^2^ = 0.770^***^	Hb:part. *R*^2^ = 0.818^***^
		K_ERY_:part. *R*^2^ = 0.063^*^		
8	Hb:part. *R*^2^ = 0.362^***^	RBC:part. *R*^2^ = 0.556^***^	Na_Ery_:part. *R*^2^ = 0.517^***^	Hb:part. *R*^2^ = 0.786^***^
	K_ERY_:part. *R*^2^ = 0.275^*^			
9	Hb:part. *R*^2^ = 0.449^***^	RBC:part. *R*^2^ = 0.394^**^	MCV_y_:part. *R*^2^ = 0.276^*^	Hb:part. *R*^2^ = 0.854^***^
	K_ERY_:part. *R*^2^ = 0.276^*^			
10	Hb:part. *R*^2^ = 0.322^***^	RBC:part. *R*^2^ = 0.494^***^	Na_Ery_:part. *R*^2^ = 0.539^***^	Hb:part. *R*^2^ = 0.798^***^
	K_ERY_:part. *R*^2^ = 0.309^*^			

**Notes.**

NA no association

* *P*-value < 0.05.

** *P*-value < 0.001.

*** *P*-value < 0.0001.

MCV mean corpuscular volume Hb hemoglobin concentration K_ERY_ erythrocyte K concentration PCV packed cell volume ICC intracellular cation concentration (erythrocyte) ECC extracellular cation concentration (plasma) DIFF difference of ECC and ICC (erythrocyte)

**Table 3:** Results of the correlation analysis of MCV, KERY, RBC, Hb and plasma [Fe] stratified by week of 30 healthy Holstein-Friesian Calves over the first 10 weeks of age.

A	MCV	RBC	MCV	Plasma [Fe]	Hb
B	K_ERY_	Hb	Hb	K_ERY_	K_ERY_
Week					
1	NA	*r* = 0.87^***^	NA	*r* = 0.38^*^	*r* = − 0.45^*^
2	NA	*r* = 0.90^***^	NA	NA°	*r* = − 0.37^*^
3	NA	*r* = 0.89^***^	NA	NA°	NA
4	NA	*r* = 0.87^***^	NA	*r* = 0.50^*^	NA
5	NA	*r* = 0.86^***^	NA	NA°	NA
6	*r* = − 0.54^*^	*r* = 0.84^***^	NA	NA°	NA
7	*r* = − 0.50^*^	*r* = 0.79^***^	NA	NA	NA
8	*r* = − 0.52^*^	*r* = 0.75^***^	NA	NA°	NA
9	*r* = − 0.53^*^	*r* = 0.63^**^	*r* = 0.50^*^	NA°	*r* = − 0.50^*^
10	*r* = − 0.56^*^	*r* = 0.70^***^	*r* = 0.42^*^	NA	*r* = − 0.49^*^

**Notes.**

*r* pearson correlation coefficient between value A and value B a *P* value of < 0.05 was considered significant NA no significant association NA° plasma [Fe] was not measured at that sampling time

* *P*-value < 0.05.

** *P*-value < 0.001.

*** *P*-value < 0.0001.

MCV mean corpuscular volume K_ERY_ erythrocyte K concentration RBC red blood cell count Hb hemoglobin concentration plasma [Fe] plasma iron concentration

Apart from a positive correlation with K_ERY_ in the first and fourth week (Table 3) no other significant association with plasma [Fe] could be identified.

The stepwise regression analysis with K_ERY_ as dependent variable including all sampling times did not reveal associations with any of the included parameters (Table 2). The regression analysis with PCV as dependent variable only identified a significant association with Hb (partial *R*^2^ = 0.368; *P* < 0.0001) (Table 2).

## Discussion

The longitudinal observation of the hemogram and blood biochemical parameters over the first 10 weeks of age in dairy calves showed a considerable decline of erythrocyte volume occurring concomitantly with a marked decline of the erythrocyte K content, and a more moderate increase of the erythrocyte Na content. These results are in line with previously published values for MCV in calves of different age, and seem to be independent of breed, gender, or whether calves are fed milk replacer or whole milk (Benesi et al., 2012; Brun-Hansen, Kampen & Lund, 2006; Egli & Blum, 1998; Holman, 1956; Mohri et al., 2004; Mohri, Sharifi & Eidi, 2007; Moosavian, Mohri & Seifi, 2010; Tennant et al., 1973).

The observed decline in MCV is consistent with the development of microcytosis, which, if associated with hypochromia, is considered a hallmark of iron deficiency anemia. Because the reduction in red blood cell volume was accompanied by a more pronounced increase of the RBC, the plasma Hb increased over time. There was thus no indication of developing anemia in this study. These results show that a declining MCV in neonatal calves is a physiological process and should thus not per se be interpreted as sign of masked iron deficiency anemia. This conclusion is supported by the plasma [Fe] determined in this study that were above concentrations previously reported in neonatal calves and indicate that there was no sideropenia in study animals in the time range during which the decline of MCV occurred (Bostedt, Jekel & Schramel, 1990; Mohri et al., 2004; Mohri, Sharifi & Eidi, 2007).

The comparably high values of plasma [Fe] at the first sampling time may be attributed to the oral administration of an iron supplement on the first day of life. A similar observation was made in an earlier study (Kume & Tanabe, 1996). It should however be noted that the single dose of 230 mg Fe^3+^ administered orally is low compared to the recommend dose of 1,000 mg of Fe^3+^ per calf to be administered parenterally (Bostedt et al., 2000; Steinhardt & Thielscher, 2003). The declining plasma [Fe] during the first weeks of life reaching a nadir at seven weeks of age, and the following increase over the last weeks of the study suggest that MR as currently fed under field conditions, containing 100 mg Fe/kg DM, as in this study provides less Fe than what healthy calves consume after weaning.

The plasma [Fe] showed a remarkable intra- and interindividual variation, and although the decline in plasma [Fe] and MCV occurred in the same time interval the plasma [Fe] did not associate to any of the parameters of the hemogram. A correlation of plasma [Fe] with Hb has been reported with considerably lower plasma [Fe] but seems to vanish with higher plasma [Fe] (Wittek, Köchler & Mader, 2014). From our results we conclude that the important decline in plasma [Fe], reaching a mean nadir of just above 20 µmol/L at seven weeks of age did not result in measurable negative effects on hematological parameters. Because Fe is also required for myoglobin production and oxygenation of muscle fibers, effects on muscle growth and function must also be considered when assessing adequacy of the dietary Fe supply.

Normal values for hematology parameters of newborn calves published in earlier studies are inconsistent which seems attributable to differences between studies in gender, breed, sample size, feeding management and analytical techniques employed. The increase in Hb and RBC observed in the present study is consistent with values determined in iron supplemented calves (Mohri et al., 2004; Moosavian, Mohri & Seifi, 2010) but are also in the range of values determined in unsupplemented calves (Brun-Hansen, Kampen & Lund, 2006; Mohri, Sharifi & Eidi, 2007; Moosavian, Mohri & Seifi, 2010). The steady PCV during the first weeks of age described here is in agreement with some earlier studies (Benesi et al., 2012; Brun-Hansen, Kampen & Lund, 2006; Mohri et al., 2004; Panousis et al., 2018), while others report a considerable decline of the PCV over the first weeks of age (Egli & Blum, 1998; Holman, 1956; Tennant et al., 1973). Similarly the slight decline of the MCH matches some of the existing reports (Benesi et al., 2012) while other authors report stable values (Panousis et al., 2018; Tennant et al., 1973) or more pronounced changes (Mohri, Sharifi & Eidi, 2007). Constant MCHC values during the first weeks of age, as in this study have been reported (Holman, 1956; Panousis et al., 2018), as have increasing (Mohri et al., 2004) and decreasing MCHC values (Mohri, Sharifi & Eidi, 2007).

The intracellular osmotic and oncotic pressure, and the osmotic equilibrium between intra- and extracellular space are important determinants of intracellular volume. Osmotic forces are primarily driven by electrolytes, carbohydrate- and other metabolites that are present on both sides of the cell membrane (Lang et al., 1998). The electrolytes most relevant for the extracellular osmotic pressure are sodium followed by chloride, while the intracellular osmotic pressure strongly depends on the intracellular [K] and to a lesser degree the [Na]. Since erythrocytes lack cell organelles requiring or producing metabolites with osmotic activity, hemoglobin is the quantitatively most abundant protein inside the red blood cell that strongly influences the intracellular oncotic pressure and thereby the cell volume (Bogner et al., 1998; Bogner et al., 2002; Lang et al., 1998). These effects reflect in the associations of the MCV with K_ERY_, DIFF and hemoglobin reported in this study.

K_ERY_ and Na_ERY_ underwent important changes over time, with K_ERY_ declining by over 60% during the study while Na_ERY_ more than doubled over the same period. Erythrocytes with different physiological [K] have been recognized in various species, and have been referred to as high potassium (HK) and low potassium (LK) phenotypes, depending on the physiological range in the specific individuum (Israel et al., 1972; Komatsu et al., 1980). Our results corroborate the findings of an earlier study based on single measurements in Holstein-Friesian calves of varying age and covering an age range of six months (Israel et al., 1972). Over the same time period only numerical decreases of the plasma [K] and a concomitant numerical and equimolar increase of the plasma [Na] were observed, with both electrolyte concentrations in plasma remaining within the reference ranges for calves of the studied age group (Dillane et al., 2018). As a result of the inverse changes in [Na] and [K] of the intra- and extracellular space, the calculated values ECC, ICC and DIFF remained constant, indicating that the observed changes occurred without measurably disturbing the osmotic equilibrium on the erythrocyte membrane. Interestingly, correlation- and regression analyses revealed consistent negative associations between MCV and K_ERY_ from the sixth week of age on, so at a time when both values approached levels of adult animals but not earlier. This finding indicates that the decline in red blood cell volume, although occurring simultaneously with changes in intracellular electrolyte concentrations is not causatively related to these.

The described changes in intracellular electrolyte content have been attributed to a reduction in the activity of the Na^+^-K^+^-ATPase (Bogner et al., 2002; Israel et al., 1972; Tosteson, 1966). A reduced activity of the Na^+^-K^+^-ATPase with a pumping ratio of potassium to sodium of 3:2 also provides a plausible explanation for the higher degree of variation observed for Na_ERY_ compared to K_ERY_ in this study (Post & Jolly, 1957).

It remains to be understood why considerably higher concentrations of erythrocyte K in neonatal erythrocytes do not seem to affect cell volume, while lower concentrations in adult erythrocytes do. Also, the purpose of this switch from HK to LK in the first weeks of age remains to be elucidated. As cell volume has a regulatory effect on a variety of cell functions, a possible explanation could be the concomitantly occurring switch from fetal to adult Hb in calves in the studied age range (Blechner, 1960; Lang et al., 1998). Studies conducted on human erythrocyte populations that were affected by mutations in the hemoglobin chain revealed that different hemoglobin morphologies influence the ion transport activity through the cell membrane by altering the K–Cl—cotransport, which affects the erythrocyte K content (Olivieri et al., 1992). After birth the fetal hemoglobin fraction diminishes and is entirely replaced by adult hemoglobin by eight to 13 weeks of age. This process is accompanied by the development of a new, adult, erythrocyte population, replacing the fetal population (Frei, Perk & Danon, 1963; Grimes, Duncan & Lassiter, 1958). In the present study the decline of fetal- and appearance of adult hemoglobin was not studied, which precluded the possibility to investigate the association between the change in type of hemoglobin and the change in MCV occurring in early life.

The available literature suggests that not all adult cattle display the LK erythrocyte phenotype. For bovines the erythrocyte phenotype has been suggested to vary between breeds (Table 4). LK and HK erythrocyte phenotypes have also been described in goats (Blechner, 1960; Evans & Phillipson, 1957), sheep (Blechner, 1961; Evans, 1957) and non-ruminant species (Beilin et al., 1966; Kerr, 1937). Studies conducted in Holstein-Friesian cattle as in the present study are scarce, but from available data it becomes apparent that the LK phenotype is at least highly predominant, and even the absence of HK phenotype in this breed has been suggested (Israel et al., 1972; Komatsu et al., 1980). Therefore, the consistent shift from a HK to the LK erythrocyte phenotype observed in our study population exclusively consisting of Holstein-Friesian calves appears plausible.

**Table 4:** Red blood cell potassium content in different breeds of cattle defined as high potassium (HK) or low potassium (LK) type erythrocyte.

Author	Species	Breed	Age	*n*	Published values (classified as LK or HK)
Evans & Phillipson (1957)	Cattle	Ayrshire	Milking	14	11.7–38.5 mEq/L RBC (**LK**)
Komatsu et al. (1980)	Cattle	Japanese Black	N/A	8	41.2 ± 2.3 mEq L RBC (**HK**)
				422	13.9 ± 0.2 mEq/L RBC (**LK**)
		Japanese Brown	N/A	5	34.8 ± 1.6 mEq/L RBC (**HK**)
				135	16.5 ± 0.3 mEq /L RBC (**LK**)
		Japanese Polled	N/A	6	39.1 ± 2.8 mEq/L RBC (**HK**)
				100	13.4 ± 0.4 mEq/L RBC (**LK**)
		Holstein	N/A	42	20.6 ± 0.5 mEq/L RBC (**LK**)
		Jersey	N/A	2	40.8 ± 0.5 mEq/L RBC (**HK**)
				27	20.5 ± 1.2 mEq/L RBC (**LK**)
Ellory & Tucker (1970)	Cattle	Jersey	N/A	13	>78 mmol/L RBC (**HK**)
				78	<62 mmol/L RBC (**LK**)

The variability of the erythrocyte phenotypes reported in the literature as well the changes occurring in K_ERY_ in early life present important caveats when considering the use of erythrocytes as easily accessible and processable cells to assess the total body- or intracellular K homeostasis, as this has been proposed in other species (Muylle et al., 1984). The results presented here suggest that erythrocytes would be particularly unsuitable as target cells to assess the K balance in the first 10 weeks of age, which can be of interest for example when treating calves with neonatal diarrhea (Golbeck et al., 2018).

As the objective of the study presented here was to describe physiological changes as the calf grows, particular care was taken to limit the bias by disease or unphysiological conditions on the investigated parameters to a minimum by closely monitoring study animals for early signs of clinical disease. Oral iron supplementation in moderate amounts at birth was included to limit hematological disturbances attributable to possible latent iron deficiency. The initially relatively high plasma [Fe] indicate that this objective was met. We therefore consider observed changes to be physiological developments corresponding to the age period of Holstein-Friesian calves.

## Conclusions

Several important changes in erythrocyte phenotype and biochemical composition occur in bovine erythrocytes in the first 10 weeks of age. Observed changes in cell volume could neither be associated to the decline in erythrocyte K content nor the plasma [Fe], leaving the underlying mechanism behind cell volume reduction unidentified. The results presented here emphasize that the sole reduction of red blood cell volume in early life cannot be interpreted as symptom for iron deficiency anemia without taking into consideration the concomitantly occurring physiological changes. The important changes of erythrocyte K content occurring in bovine neonates make red blood cells particularly unsuitable for the reliably representation of the K content of the intracellular space.

##  Supplemental Information

10.7717/peerj.7248/supp-1 Figure S1 Feed intake of 25 neonate heifer calves during the first 80 days of age Data presented as mean (+SD). Up to day 10 values represent intake of acidified whole milk. Intake of milk replacer is depicted afterwards. Feed intake of bull calves not included in this figure.

10.7717/peerj.7248/supp-2 Figure S2 ICC, ECC and DIFF in 30 Holstein-Friesian calves over the course of the first 10 weeks of age Mean (+SD) for intracellular (erythrocyte) cation concentration (**ICC**, triangles), extracellular (plasma) cation concentration (**ECC**, closed squares) and difference of ICC and ECC (**DIFF**, closed circles) in 30 Holstein Fresian calves over the course of the first 10 weeks of age (n=30 except for ICC and DIFF in weeks 4,6 and 9 with n=29, week 3 with n=28 and week 1 with n= 26). Values marked the superscript letter “a” differ significantly (P¡0.05) from the value of the preceeding week, whereas a “b” indicates a difference to the value of two weeks before.

10.7717/peerj.7248/supp-3 Figure S3 Plasma [Fe] at sampled day of life in 30 Holstein-Friesian calves over the first ten weeks of age Values for plasma Fe (µmol/l) in 30 healthy neonate calves (25 heifers (closed circles) and five bulls (asterisk)) against their respective age during the 1st (**A**), 4th (**B**), 7th (**C**; *n* = 29) and 10th (**D**) sampling time. Slope of the linear regression line, goodness of Fit ( *R*^2^) and significance of deviation from zero (P) are given.

10.7717/peerj.7248/supp-4 Dataset S1 Blood measurements in 30 neonate Holstein-Friesian calves over the first 10 week of life

10.7717/peerj.7248/supp-5 Dataset S2 Liquid feed intake of 25 neonate heifer calves over the first 10 weeks of life

## Additional Information and Declarations

### Data Availability

The following information was supplied regarding data availability: Raw data is available as Supplemental Files.
