# Supplementary figures and images for: Changes of the erythrocyte phenotype and blood biochemistry in dairy calves during the first ten weeks of age
Source: PeerJ. 2019 Jul 16;7:e7248. doi: 10.7717/peerj.7248 (PMC6640622; doi:10.7717/peerj.7248)

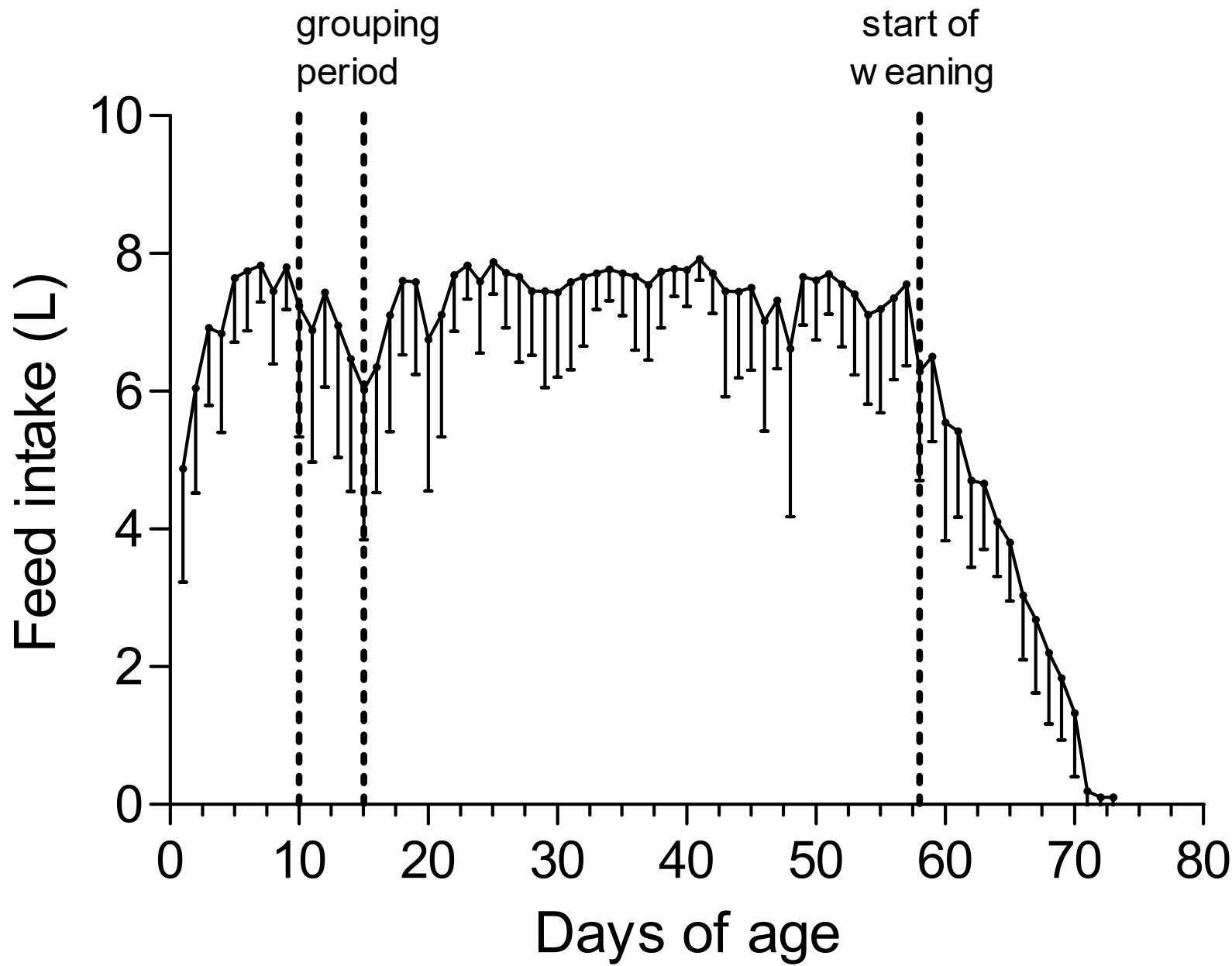

Supplement: Figure S1 — Data presented as mean (+SD). Up to day 10 values represent intake of acidified whole milk. Intake of milk replacer is depicted afterwards. Feed intake of bull calves not included in this figure. [file peerj-07-7248-s001.pdf]

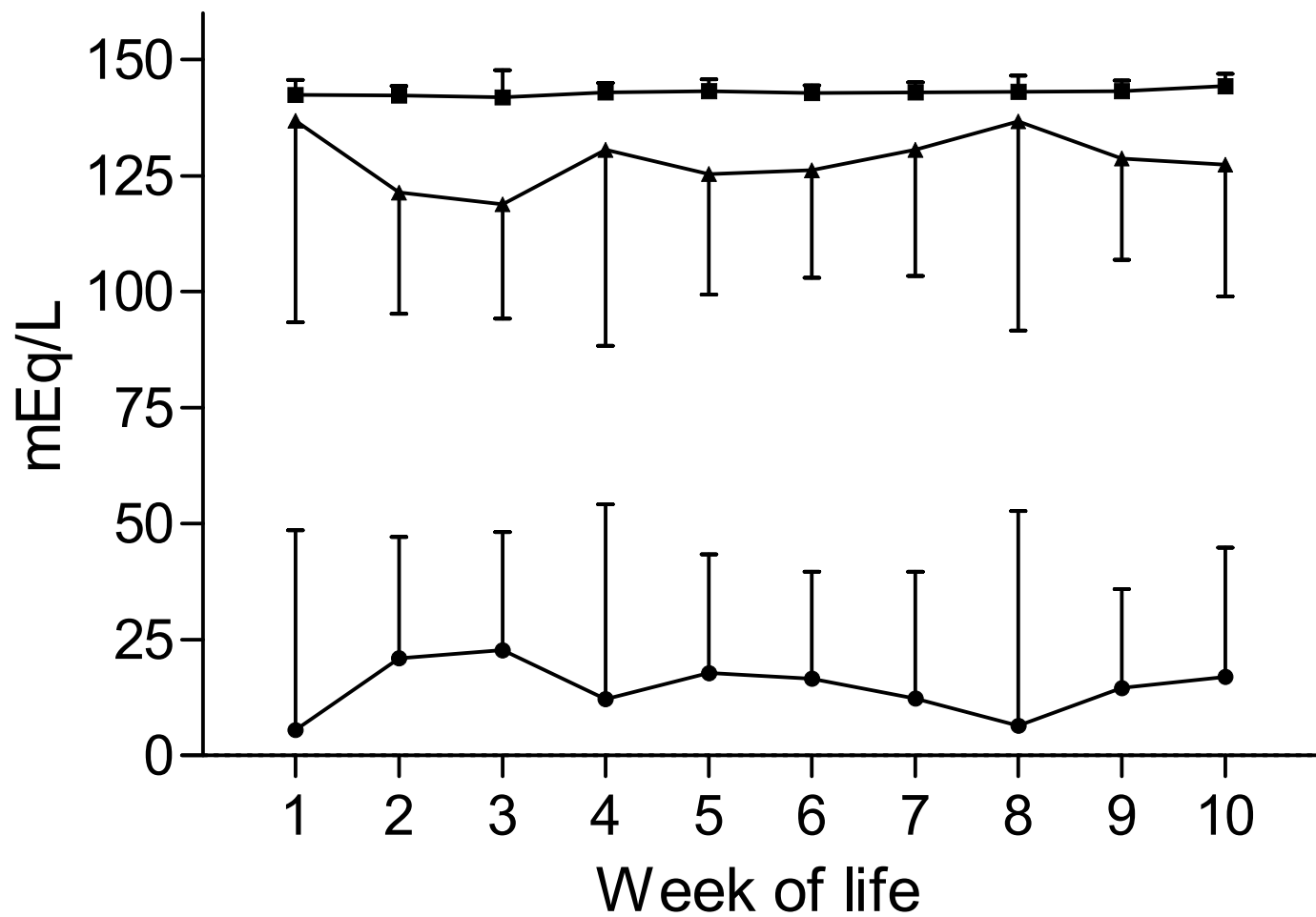

Supplement: Figure S2 — Mean (+SD) for intracellular (erythrocyte) cation concentration (ICC, triangles), extracellular (plasma) cation concentration (ECC, closed squares) and difference of ICC and ECC (DIFF, closed circles) in 30 Holstein Fresian calves over the course of the first 10 weeks of age (n=30 except for ICC and DIFF in weeks 4,6 and 9 with n=29, week 3 with n=28 and week 1 with n= 26). Values marked the superscript letter “a” differ significantly (P¡0.05) from the value of the preceeding week, whereas a “b” indicates a difference to the value of two weeks before. [file peerj-07-7248-s002.pdf]

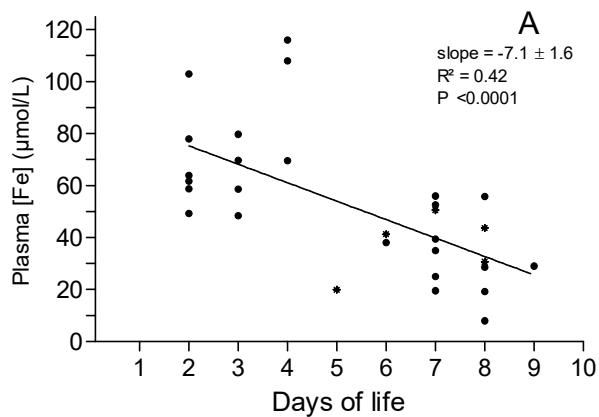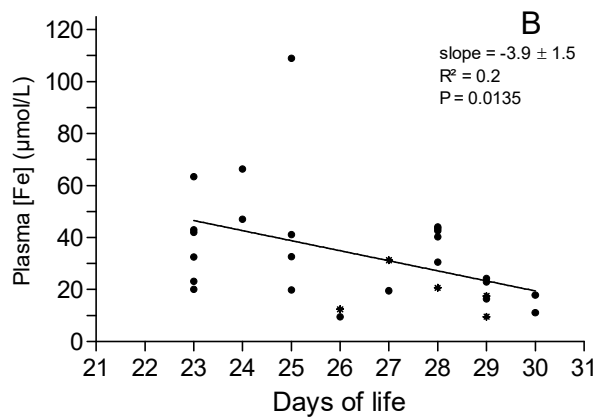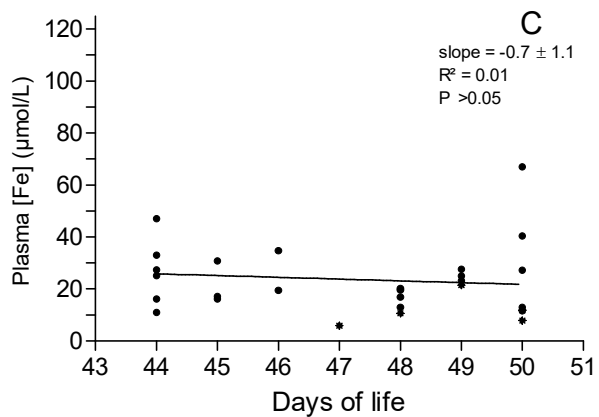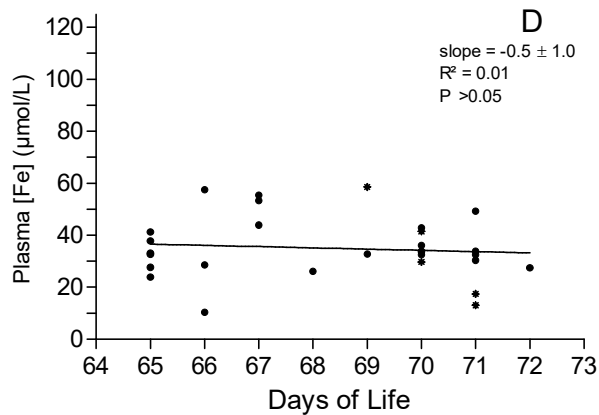

Supplement: Figure S3 — Values for plasma Fe (µmol/l) in 30 healthy neonate calves (25 heifers (closed circles) and five bulls (asterisk)) against their respective age during the 1st (A), 4th (B), 7th (C; n = 29) and 10th (D) sampling time. Slope of the linear regression line, goodness of Fit ( R2) and significance of deviation from zero (P) are given. [file peerj-07-7248-s003.pdf]
